# [Corrigendum] Electroacupuncture at GV20-GB7 regulates mitophagy to protect against neurological deficits following intracerebral hemorrhage via inhibition of apoptosis

**DOI:** 10.3892/mmr.2026.13930

**Published:** 2026-06-03

**Authors:** Ruiqiao Guan, Zhihao Li, Xiaohong Dai, Wei Zou, Xueping Yu, Hao Liu, Qiuxin Chen, Wei Teng, Peng Liu, Xiaoying Liu, Shanshan Dong

Mol Med Rep 24: 492, 2021; DOI: 10.3892/mmr.2021.12131

Following the publication of this paper, it was drawn to the Editor's attention by a concerned reader that, in comparing the bar charts shown in Fig. 2D and F on p. 5 representing the quantification of the western blot data featured in Fig. 2C and E respectively, these bar charts were strikingly similar, suggesting that the same chart may have erroneously been included in this figure twice to represent the different experimental conditions.

The authors have re-examined their original data, and realize that the bar chart that was correctly shown for Fig. 2F was erroneously duplicated in the figure to show the quantification of the data in Fig. 2D. The corrected version of Fig. 2, now showing the correct data for the bar chart in Fig. 2D, is shown on the next page. Note that this error did not affect the overall conclusions reported in the paper. All the authors agree with the publication of this corrigendum, and are grateful to the Editor of *Molecular Medicine Reports* for allowing them the opportunity to publish this. They also apologize to the readership for any inconvenience caused.

## Figures and Tables

**Figure 2.**
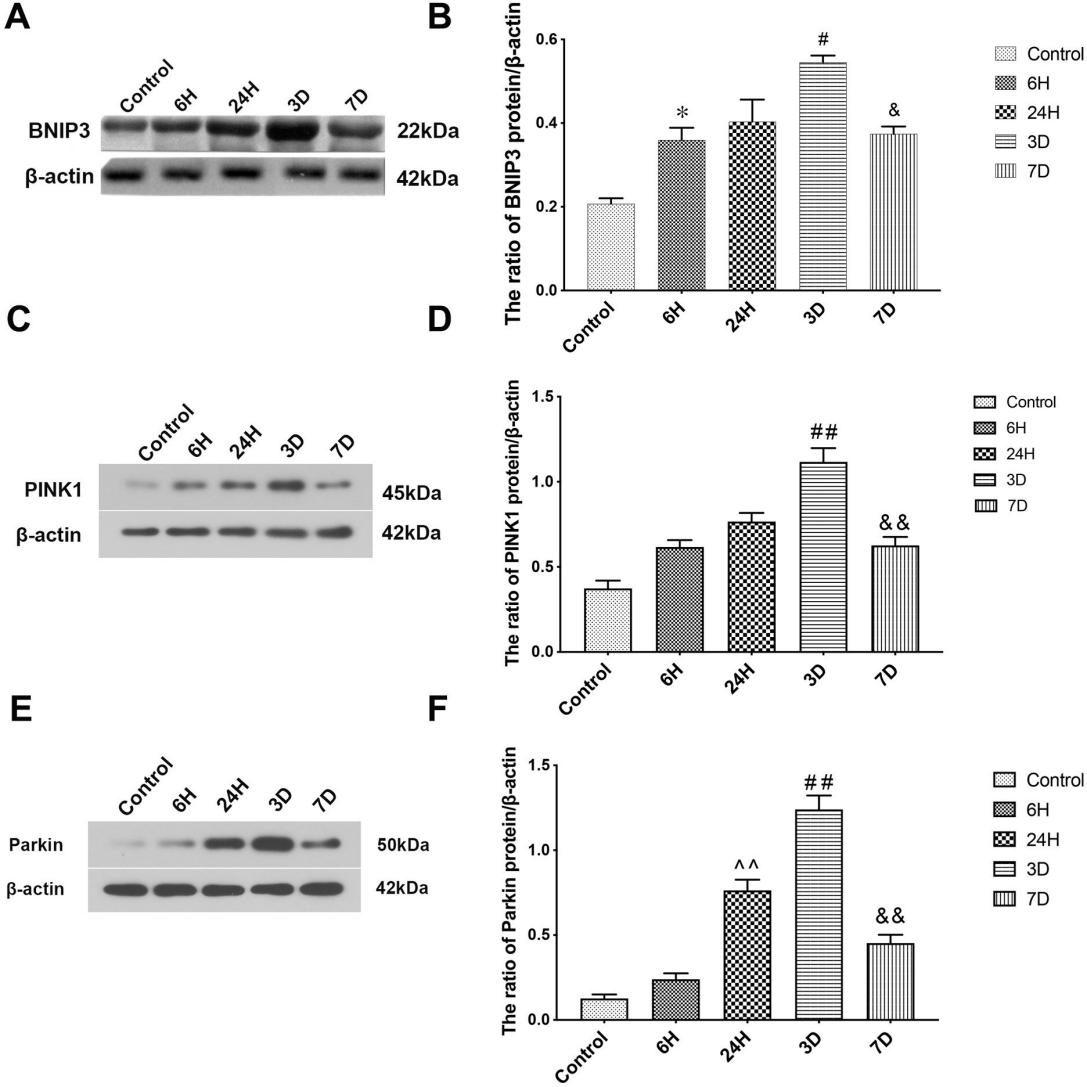
Mitophagy is activated following intracerebral hemorrhage. Expression levels and semi-quantitative analysis of (A and B) BNIP3, (C and D) PINK1 and (E and F) Parkin, as determined by western blot analysis. β-actin protein was used as the loading control. Data are expressed as the mean ± SEM (n=5/group) and analyzed by one-way ANOVA followed by Tukey's multiple comparisons test. *P<0.05 vs. Control; ^^^^P<0.01 vs. 6 h; ^#^P<0.05, ^##^P<0.01 vs. 24 h; ^&^P<0.05, ^&&^P<0.01 vs. 3 days. BNIP3, BCL/adenovirus E1B 19k Da-interacting protein 3; PINK1, PTEN-induced putative kinase 1.

